# Low-Humidity Sensing Properties of Multi-Layered Graphene Grown by Chemical Vapor Deposition

**DOI:** 10.3390/s20113174

**Published:** 2020-06-03

**Authors:** Filiberto Ricciardella, Sten Vollebregt, Tiziana Polichetti, Pasqualina M. Sarro, Georg S. Duesberg

**Affiliations:** 1 Department of Microelectronics, Delft University of Technology, 2628 CT Delft, The Netherlands; s.vollebregt@tudelft.nl (S.V.); p.m.sarro@tudelft.nl (P.M.S.); 2 Institute of Physics, Universität der Bundeswehr München, 85577 Neubiberg, Germany; georg.duesberg@unibw.de; 3 ENEA Research Center, I-80055 Portici, Italy; tiziana.polichetti@enea.it

**Keywords:** graphene, defects, humidity, chemical vapor deposition, sensitivity, sensors

## Abstract

Humidity sensing is fundamental in some applications, as humidity can be a strong interferent in the detection of analytes under environmental conditions. Ideally, materials sensitive or insensitive towards humidity are strongly needed for the sensors used in the first or second case, respectively. We present here the sensing properties of multi-layered graphene (MLG) upon exposure to different levels of relative humidity. We synthesize MLG by chemical vapor deposition, as shown by Raman spectroscopy, Atomic Force Microscopy (AFM) and Scanning Electron Microscopy (SEM). Through an MLG-based resistor, we show that MLG is scarcely sensitive to humidity in the range 30%–70%, determining current variations in the range of 0.005%/%relative humidity (RH) well below the variation induced by other analytes. These findings, due to the morphological properties of MLG, suggest that defective MLG is the ideal sensing material to implement in gas sensors operating both at room temperature and humid conditions.

## 1. Introduction

Humidity monitoring is of crucial importance in numerous applications, including industrial and pharmaceutical production process, food safety, and ecological matters. As such, many reports have focused on materials providing the best performance upon exposure to humidity, such as metal oxide semiconductors (MO_x_), porous ceramics, polymers, ceramic/polymer, electrolytes, graphene-oxide, carbon nanotubes and their composites [1,2,3,4,5,6,7,8,9,10,11]. On the other hand, among the applications concerning the gas sensing field, e.g., for environmental monitoring, humidity behaves as a strong interferent [2,12]. Under ambient conditions, e.g., in the presence of water vapors and operating at room temperature (RT), the signal detection can be largely influenced, leading to barely discriminative responses from analytes and humidity. Additionally, upon lowering the operating temperature from high values, much larger effects of air humidity are induced on the sensing performance [13,14].

Humidity is expressed as a percentage, since it is defined as a ratio of the amount of water vapor contained in air to the maximum (saturated) moisture level that the air can hold at a same given temperature and pressure. Existing as a ratio, it is also indicated as relative humidity (RH) [2]. Since humidity is an unavoidable component when working under environmental conditions, it is crucial to minimize the impact or find a route to differentiate the contributions of the diverse species.

The most explored approaches consist of engineering the architecture of the transducers, for instance, incorporating a microheater [8,15,16], or using a posteriori analysis through artificial intelligence [17,18,19,20]. A much more efficient approach could be the investigation of materials which are not particularly affected by humidity, while being sensitive to other analytes.

In this framework, here, we demonstrate that relatively defective and rough multi-layered graphene (MLG) is insensitive to humidity, while having previously proved that the same MLG is a very promising sensing material upon other analytes [21,22,23]. In those reports, we demonstrated that MLG shows a sensitivity up to ~6%–7%/ppm towards nitrogen dioxide (NO_2_). We hereby quantify how much MLG is not sensitive in the RH range 30%–70%. This range was selected in order to mimic humidity levels quite close to the standard ambient conditions.

Our choice of investigating MLG grown by chemical vapor deposition (CVD) upon RH exposure is driven by some crucial considerations. Firstly, it is well known that defective carbon-based materials are more prone to atoms and molecules adsorption which can lead to enhanced sensitivity [24,25,26,27,28,29,30,31,32,33]. Secondly, it has been shown that the response of thicker graphene films used as sensing layers can be higher than that of single layer of graphene (SLG) [24,34]. Finally, either non-defective SLG, double or few-layer graphene can behave differently among them upon water vapors, as proven by both theoretical and experimental works regarding sensing and wettability [35,36,37,38,39,40,41,42].

Therefore, we set the boundary conditions of the experiment to maximize the interaction between the species (humidity) and the sensing layer (MLG). In order to purely obtain more insight on the properties of MLG towards RH, we used the simplest transduction structure, i.e., a resistor, acting as a chemi-resistor when exposed to water vapors.

## 2. Materials and Methods

We synthesized MLG by CVD at ~1000 °C on pre-patterned Mo catalyst in an AIXTRON BlackMagic Pro tool. As a carbon precursor, we used 20 sccm of methane (CH_4_) in Ar/H_2_ atmosphere for 5 min under the pressure of 25 mbar [43].

We firstly investigated MLG through Raman spectroscopy using a Renishaw inVia Reflex (Renishaw, Wotton-under-Edge, UK) equipment used in back-scattering configuration. The tool was equipped with a 514 nm laser and a 50× objective with a numerical aperture of 0.50. We mapped a sample area of 100 × 100 μm^2^, acquiring 100 spectra at a space interval of 10 μm. To inspect the surface morphology of MLG, we used a NT-MDT NTEGRA SPECTRA (NT-MDT, Moscow, Russia) atomic force microscope (AFM) and a Philips XL50 Scanning Electron Microscope (SEM) (Philips, Amsterdam, The Netherlands). AFM operated in tapping mode with an n-doped Si NSG10 tip, acquiring images with 512 lines and rate of 0.60 Hz. SEM operated with a beam acceleration voltage of 15 kV.

To fabricate the devices, we adopted the transfer-free process. That process, described in detail in a prior report [43], consists of growing MLG on top of Mo layer which was previously pre-patterned by lithographic steps and dry etching. After the growth, the catalyst layer beneath the MLG was removed by wet etching, causing the MLG to drop on the SiO_2_/Si substrate on the pre-defined locations and circumventing any transfer step of MLG to a different target substrate. MLG was contacted with Cr/Au (10/100 nm) electrodes deposited by e-beam evaporation in combination with a lift-off process in N-Methyl-Pyrrolidone (NMP). To analyse the electrical properties of the fabricated MLG-based resistors, we measured the current–voltage (I–V) characteristic using a semi-automatic probe-station equipped with an Agilent 4156C semiconductor parameter analyser.

We exposed the devices to water vapors in a customized Gas Sensor Characterization System (GSCS) comprising a stainless-steel chamber (40 cl) placed in a thermostatic box and provided with an electrical grounded connector for bias and conductance measurements. Temperature and pressure were fixed at ambient conditions, (22 ± 2) °C and (1.00 ± 0.05) bar, while RH level was varied according to the protocols described later. During the measurements, we biased the resistor at constant DC voltage equal to 1 V with a Precision Power Supply TTi QL355 T. The current values were recorded by a high-resolution pico-ammeter (Keithley 6485).

We implemented three different protocols simulating the variation of RH levels.

Test1 consisted of a cycle having three ramps of RH variation. In the first ramp, RH level was increased from 50% up to 70%. It was followed by a ramp down of the RH level, from 70% down to 30%. In the third part, RH level returned to the initial value of 50%.

Similar to Test1, Test2 consisted of three stages, although the verse of the ramps is reversed compared to Test1.

Test3 consisted of a double repetition of Test1 to address the reproducibility of the performances.

In all of these protocols, RH level varied with 5% step every 10 min in both ramp up and down.

## 3. Results and Discussion

**Figure 1:**
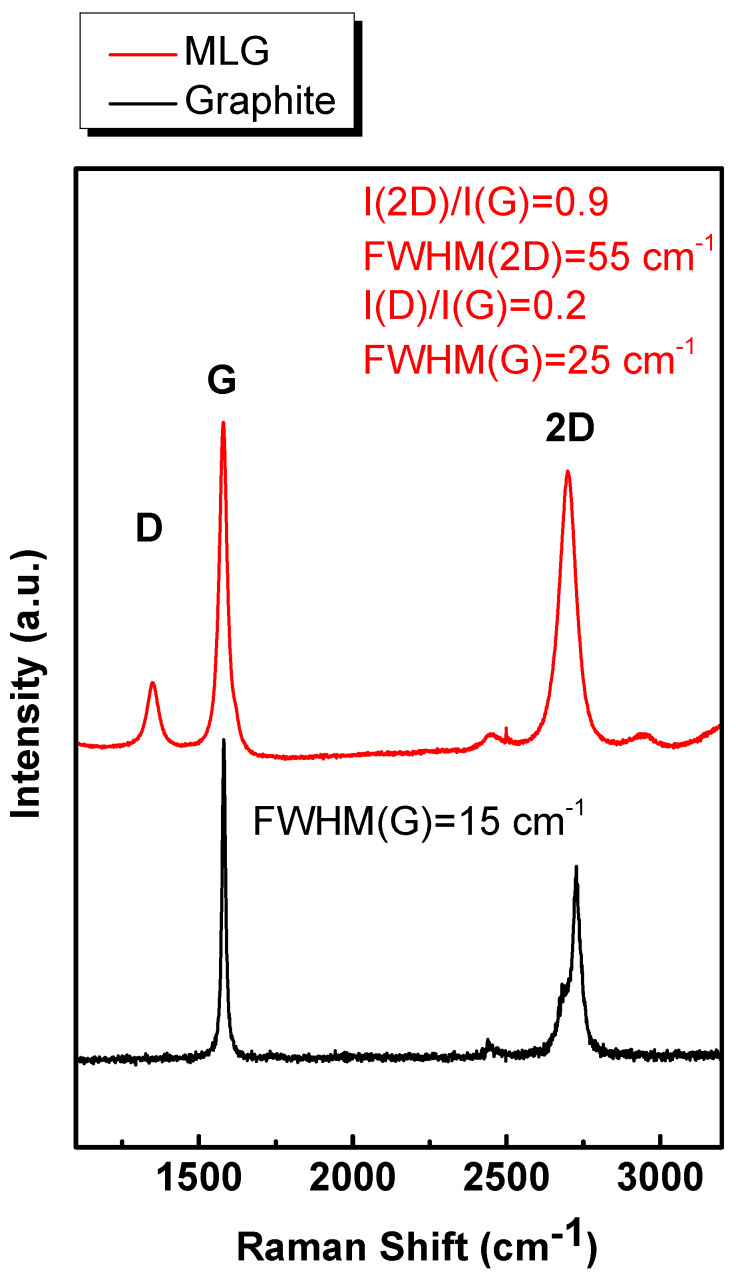
Raman spectra of multi-layered graphene (MLG) grown by chemical vapor deposition (CVD) (red line) and highly oriented pyrolitic graphite (black line). Both spectra are normalized to the G band.

Figure 1 displays the Raman profile of the grown material (red line) averaged from 100 spectra (Figure S1). As a comparison, the spectrum of the graphite (GR) (black line) is reported.

The most evident differences between the two spectra concern the band attributed to both the disordered mode (D ~1350 cm^−1^) and the overtone of the D line (2D) at ~2700 cm^−1^ [44]. The rise of the D-band indicates the presence of some defects in the grown material. The 2D-band in the red profile, having FWHM(2D) ~55 cm^−1^, is distinctly sharp without any shoulder at lower wavenumbers, as conversely seen in the GR’s spectrum, indicating the formation of turbostratic MLG [44,45].

Analyzing the other features of the spectra in more detail, we infer that the full-width at half maximum (FWHM) of the C-C related line G (~1580 cm^−1^) passes from ~15 cm^−1^ in GR to ~25 cm^−1^ in the MLG. The variation of FWHM(G) suggests that the grown material differs significantly from GR. The nature of the MLG as being grown is definitively assessed by the value of I(2D)/I(G), i.e., the ratio between the intensity of 2D and G bands, being the ratio correlated to the number of layers [44,46,47]. For GR, I(2D)/I(G) ~0.6 while for MLG I(2D)/I(G) ~1.

**Figure 2:**
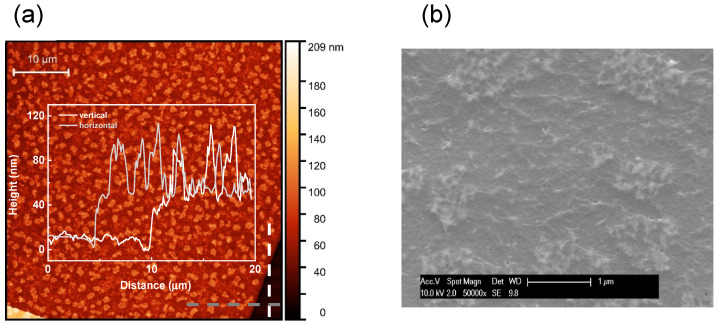
(**a**) Atomic force microscopy (AFM) topography of an MLG scanned area of 2500 μm^2^. The step-height profiles are measured along the dashed lines vertically (white) and horizontally (grey) drawn in the bottom-right corner of the image. (**b**) Scanning electron microscopy (SEM) image of MLG.

To further address the nature of the multi-layered structures, we performed AFM and SEM analysis (Figure 2). Figure 2a shows the AFM topography of the scanned area. Figure 2b displays the captured SEM image.

**Figure 3:**
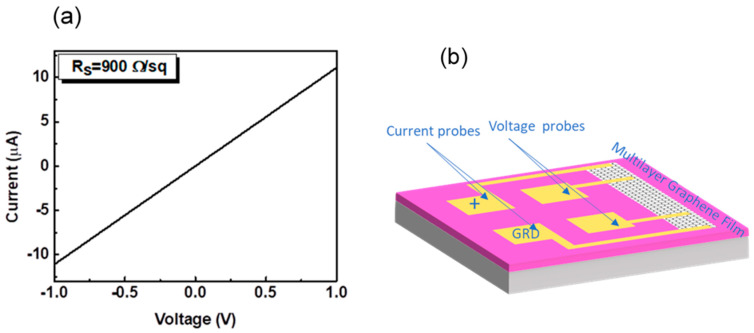
(**a**) Current–voltage (I–V) characteristic of the MLG-based resistor. (**b**) Schematic of the device. The resistor consists of a bar having length and width, between the voltage probes, of 206 and 2 μm, respectively.

The profiles (Figure 2a) display step-heights of about 40 nm. The step value is measured from SiO_2_ surrounding the MLG-based bar (Figure 3). The SiO_2_ film, however, presents an off-set of ~20–30 nm, since the plasma etching used when patterning the Mo layer typically etches 20–30 nm of SiO_2_ due to the limited selectivity of this etch step. The film of SiO_2_ is therefore thinner than the film initially deposited (90 nm) and the thickness of MLG is lower than that was measured with AFM (Figure S2). The estimated thickness of MLG is around 10 nm, in agreement with the results previously reported, obtained by UV-Vis [48]. The thickness could indicate a structure more similar as thin graphite. Of note, in other works [27,49], as well as through the Raman analysis, we have proved that the grown material consists of MLG rather than thin graphite [50]. Figure 2a shows jagged surface of MLG with a roughness of ~3 nm [49]. The jagged surface is further attested by the SEM image (Figure 2b). Figure 3a reports the I–V curve of the device based on MLG, depicted in Figure 3b.

**Figure 4:**
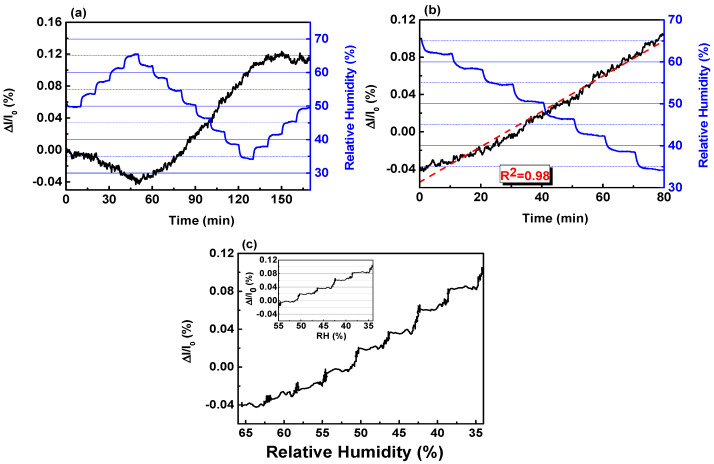
(**a**) Real-time behavior of the current variation (black line) upon Test1 (blue line). (**b**) Close-up of the output extracted from panel (**a**) while decreasing RH level (blue line). The dashed red line represents the fit of the current variation (black line). (**c**) Current behavior plotted as a function of decreasing RH level instead of as a function of time like in panel b. The scale of *x*-axis in panel (**c**) is reversed for the sake of clarity with the graph in panel (**b**). Inset. Zoom on the current plot shown in the main panel.

The linearity of the I–V curve testifies that the device based on MLG is a resistor. Afterwards, we tested the resistor upon Test1 (see Section 2) and the behavior of *(I − I*_0_*)/I*_0_ is reported as a function of time in Figure 4, where *I*_0_ and *I* are the values of the current recorded at the inlet and along the exposure at the water vapors, respectively.

We observed the tendency of the conductance to be reduced (increased) while increasing (decreasing) the amount of water vapors injected in the chamber. The oxidizing trend towards H_2_O is well-aligned with the findings reported elsewhere [36,51], despite the fact, in those cases, the sensing layer was synthetized through liquid phase exfoliation (LPE) or mechanical exfoliation, differently from the CVD route used in this report.

Under the successive H_2_O exposure steps (Figure 4, blue line), the signal reported as a function of time (black line) never reaches a plateau along the exposure time of 10 min. On the contrary, the variation of the current shows a robust linear dependency from the humidity, as proven by the value of R^2^ equal to 0.98 in the range 60–30% of RH (Figure 4b). Considering the two extreme RH levels, we could estimate the sensitivity (S), defined as the minimum input of %RH that can induce a detectable change in the output [52]. The value 0.5 nA/% was determined as *(I*_60%_ *− I*_30%_)/30%, where *I*_60%_ *(I*_30%_*)* is the current recorded when the RH level is 60% (30%) (Figure S3). The sensitivity expressed as a percentage (~0.005%/%RH) (Figure 4b) suggests that MLG is rather scarcely affected by the adsorption of water molecules, differently from what occurs towards other analytes. In previous reports, we have demonstrated that MLG is three orders of magnitude (~6%–7%/ppm) more sensitive towards NO_2_ compared to RH [23]. MLG has also higher sensitivity upon ammonia (NH_3_) (~0.01%/ppm) than RH, provided that our material is not such affine to NH_3_ [22].

The scarce sensitivity upon water molecules is further strengthened by the analysis of the output in the other two non-linear zones (Figure 4a). Increasing the RH level of 15%, from 50% up to 65% or from 35% up to 50%, determines either the variation of only 0.04% or the substantial stationarity of the current. We plotted the transient reported in Figure 4b as a function of RH level (Figure 4c) to better highlight the effects of RH. It can be seen that down to RH = 50%, the variation is almost linear with humidity. At lower values of RH, step-like variations of Δ*I*/*I*_0_ start appearing with heights of about 0.02%/5% (Inset).

To ensure that the achieved conclusion is not dependent on the specific executed protocol, we conducted a measurement (Test2) which is slightly different from Test1 (see Section 2). Through Test2, once more we showed the linear dependency of Δ*I*/*I*_0_ by RH level with a correlation coefficient R^2^ = 0.98 while varying RH levels from 35% up to 65%. The estimated sensitivity was equal to 0.4 nA/% which, as a percentage, corresponds to 0.004%/%RH (Figure S4). The output, reported as a function of RH level, showed step-like variations having maximum heights of 0.004%/%RH. The findings support the achievements reported in Figure 4 and straightforwardly bring us to conclude that the grown materials shows no strong response to H_2_O molecules.

**Figure 5:**
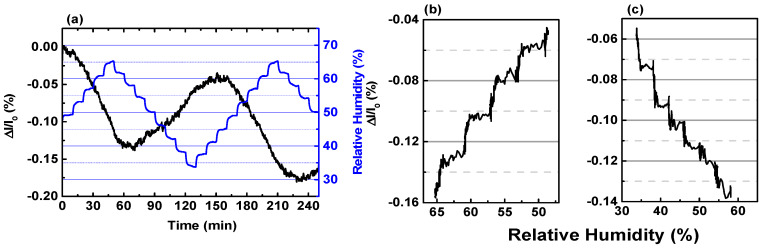
(**a**) Real-time behavior of the current variation upon exposure to Test3. (**b,c)** Close-up of the output extracted from panel (**a**) while decreasing or increasing the relative humidity (RH) level (blue line). In panel (**b**), the scale of the *x*-axis is reversed for the sake of clarity with the graph in panel (**a**).

As the executed protocol did not show any effect on the trend of the conductance, we finally tested the reproducibility of the performances of MLG upon subsequent RH variations. Figure 5 displays the transient recorded while applying Test3 (see Section 2).

The graph in Figure 5a provides evidence of the behavior previously described, showing reduced (increased) conductance while increasing (lowering) the amount of the blown water vapors. Fitting the signal during the ramps down and up of the RH levels, we observed once more the robust linear dependency from the RH level, as confirmed by the values of R^2^, equal to 0.99 and 0.96, respectively (Figure S5). From the two linear zones, the sensitivity was estimated to be roughly 0.01 and 0.008%/%RH during the ramp down and ramp up of the protocol, respectively (Figure S5). The output, as a function of RH levels, shows a maximum step-like variation of about 0.03%/%RH (Figure 5b,c). The values of S as well as the step-like variations are in close agreement with the results of the previous tests, addressing the reproducibility of the performances of MLG, as confirmed by the results obtained by a second MLG-based device (Figure S6). More importantly, the findings definitively prove the scarce reactivity of the grown MLG to the water vapors, in spite of the most suitable conditions used for the humidity detection, i.e., thick and jagged material, as reported in Table 1. It can be seen that the sensitivity upon RH increases with the thickness and defectivity of the material.

In a previous publication, it has been reported that the scarce reactivity to RH can be ascribed to the high conductivity of graphene [52]. In our case, we can surely exclude this explanation, provided that MLG hereby adopted is rather resistive, presenting R_s_ in the range of 1 kΩ/sq [55]. We also can exclude the effects of the less adsorption sites due to the polymer residues originating from the transfer of MLG to the target substrate [56]. Adopting the transfer-free process detailed in Materials and Methods, we avoid any transfer step from the growth substrate and we typically do not observe substantial difference between the Raman spectra before and after the lift-off process.

As stated by Popov and co-workers, different conductive centers contribute to the adsorption of the water molecules [24]. Depending on the components dominating the adsorption process, the conductivity can be enhanced or lowered [54]. In case of MLG films, a higher capture cross-section is realized if the edge defects govern the adsorption of the water molecules. Due to the ionic conductivity, the conductive chains are formed on the hydronium ions (H_3_O^+^). Should the chains form a percolation network, the increase of the conductivity could be induced [11,24]. We speculate that not only a single explanation but a combination of effects can be sought to justify the scarce reactivity of MLG in the humid environment [57]. Fan et al. already proved that double layer graphene is less sensitive than SLG upon H_2_O exposure, indicating that a thicker sensing material can have less reactivity towards H_2_O [38]. The multi-layered and turbostratic nature of our material can also justify the increase of the conductance compared to the opposite trend reported in ref [38].

The thickness of MLG (~10 nm) can also be responsible of other phenomena. Smith at al. have reported that the interaction between the electrostatic dipole moment of the water and the impurity bands in the SiO_2_ substrate determines the electrostatic doping of SLG, with this effect being reduced for double layer graphene [39]. For MLG, the large electric field (in the range of 10^9^ V/m) reported for SLG [58] could be substantially reduced, determining none or lower interaction with the SiO_2_ substrate. Moreover, we have previously shown that the molecules of the analytes are adsorbed by both edges and basal planes of the CVD-grown MLG films [27]. In that paper, the conductance of the MLG-based device shows an overall similar trend while exposed humidity vapors, confirming the acceptor-like nature of H_2_O like NO_2_ [36,51]. The CVD growth process can induce some intrinsic defects, due to the reconstruction of the lattice in non-hexagonal rings, namely pentagons, hexagons and heptagons [26,59,60]. It is very likely that such defects, known as Stone–Wales defects and intrinsically ascribed to the CVD process, as well as edge defects are responsible of the weak and slow reactivity upon RH, while enhancing the reactivity upon other analytes [22,23,27]. This conclusion is strengthened by comparing the results with a previous work, where MLG was synthesized by LPE and deposited by drop-casting or through Langmuir−Schaefer technique [51,61]. In both cases, the defects originating from CVD were not present evidently. The material synthesized by LPE differed morphologically from the CVD-grown one, presenting only edge defects due to the dimension of flakes, thus resulting in a lower conductivity [62] and quite different behavior of the conductance. The binding energy at the edge sites dominates over basal plane sites and induces the conduction through the edges. The contribution of the edges defects can also induce an opposite behavior towards the analytes, as observed by Nufer et al., where a p-type dopant, such as acetone, determined the increase of resistance [61].

Using CVD-grown MLG for gas sensing applications under ambient moisture conditions guarantees that the interaction between the sensing layer and the analyte is the main contribution, with rather negligible effects provided by the humidity. Such a scarcely sensitive material upon a strong interferent, such as water, is ideal for developing gas sensors operating under environmental conditions, since it enables a sort of a priori discrimination of the signal based on the morphological properties of the sensing material.

## 4. Conclusions

We analyzed the sensing properties of MLG upon exposure to vapors of water. We observed the overall tendency of the water to behave as an acceptor-like analyte, inducing the lowering (increase) of the conductance of the MLG-based chemi-resistor while increasing (decreasing) the amount of the vapors. Along the range 30%–70% of RH, we showed a current variation as low as 0.005%/%RH, definitively confirming the scarce sensitivity of MLG upon humidity. We attributed such low sensitivity to the thickness and morphological structure of the material. The presented outcomes suggest interesting applications of MLG grown by CVD in gas sensing applications under environmental conditions.

## Figures and Tables

**Table 1:** Sensitivity of graphene-layers upon RH exposure. Only resistive sensors were considered to fairly compare our results.

Material	Sensitivity (%/%RH)	Reference
CVD SLG	0.3–1.3	[35]
CVD BLG	29	[53]
FLG ^1^	93	[37]
CVD MLG	7–27	[24]
PECVD MLG ^2^	8–35	[54]
CVD MLG	0.005	(this work)

^1^ FLG (Few-Layered Graphene) were synthesized by the arc-discharge method. ^2^ PECVD stands for Plasma Enhanced CVD.

## Supplementary Materials

The following are available online at https://www.mdpi.com/1424-8220/20/11/3174/s1, Figure S1: Collection of 100 Raman spectra acquired on MLG grown by CVD; Figure S2: Sketch of the plasma etching step during the transfer-free process; Figure S3: Current behavior of MLG-based chemi-resistor upon Test1; Figure S4: Transient behavior of Δ*I/I*_0_ upon Test2; Figure S5: Close-up of the transient behaviors upon Test3; Figure S6: Transient behavior of Δ*I/I*_0_ upon Test3 of a second MLG-based chemi-resistor.
