# Supplementary material for: Low-Humidity Sensing Properties of Multi-Layered Graphene Grown by Chemical Vapor Deposition
Source: Sensors (Basel). 2020 Jun 3;20(11):3174. doi: 10.3390/s20113174 (PMC7313702; doi:10.3390/s20113174)
Supplement: Supplementary file 1 [file sensors-20-03174-s001.pdf]

sensors

MDPI

Communication

Low-Humidity Sensing Properties of Multi-Layered Graphene Grown by Chemical Vapor Deposition

Filiberto Ricciardella [1,2,*], Sten Vollebregt [1], Tiziana Polichetti [3], Pasqualina M. Sarro [1] and Georg S. Duesberg [2]

1 Department of Microelectronics, Delft University of Technology, 2628 CT Delft, The Netherlands; s.vollebregt@tudelft.nl (S.V.); p.m.sarro@tudelft.nl (P.M.S.)
2 Institute of Physics, Universität der Bundeswehr München, 85577 Neubiberg, Germany; georg.duesberg@unibw.de
3 ENEA Research Center, I-80055 Portici, Italy; tiziana.polichetti@enea.it
* Correspondence: filiberto.ricciardella@gmail.com or filiberto.ricciardella@unibw.de

Received: 28 April 2020; Accepted: 1 June 2020; Published: 3 June 2020

1. Material Analysis

Figure S1 reports the Raman spectra collected on the material grown by chemical vapor deposition as described in the main text.

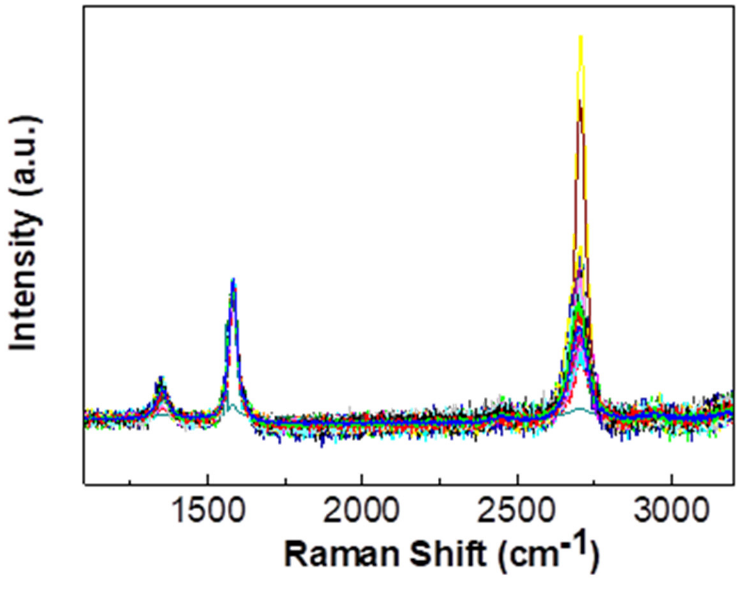

Figure 1. Collection of 100 Raman spectra acquired on multi-layered graphene (MLG) grown by chemical vapor deposition (CVD).

An area of 100 × 100 μm² was mapped, acquiring 100 spectra at a space interval of 10 μm. Figure S2 reports the sketch of the plasma etching process during the pattern of the Mo layer.

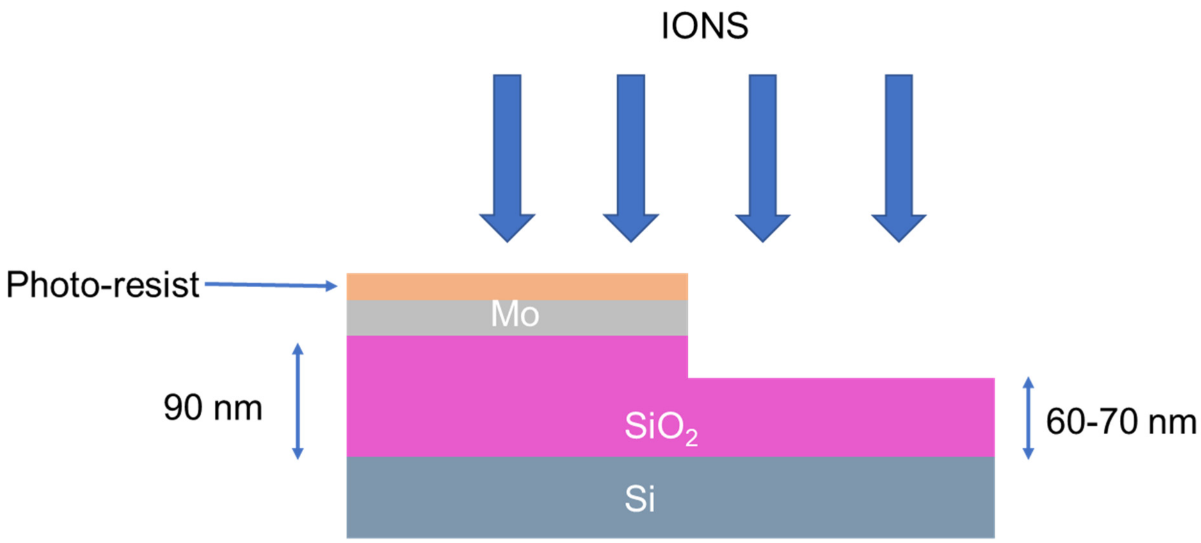

Figure 2. Sketch of the plasma etching step during the transfer-free process of MLG.

The sketch shows the effects of the ions in lowering ~20–30 nm the initial thickness of the SiO_2 film when patterning the Mo layer.

2. Analysis of Sensing Properties

Figure S3 reports the close-up of the transient of the MLG-based resistor while decreasing relative humidity (RH) level (blue line) in Test1.

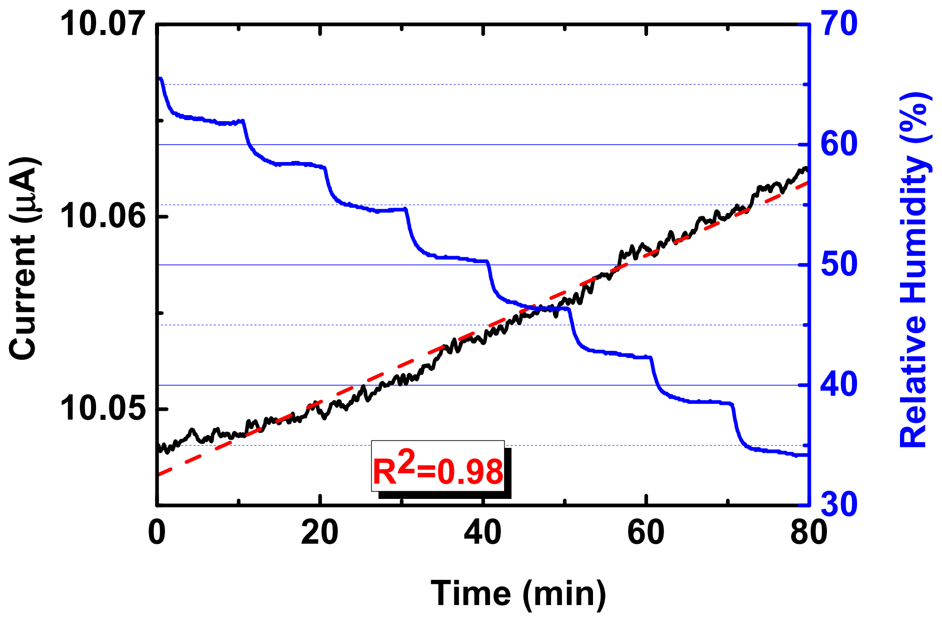

Figure 3. Close-up of the current extracted from Figure 4a (main text) before the normalization to I_0. The dashed red line represents the fit of the current (black line).

From the graph in Figure S3, we determined the sensitivity (0.5 nA/%) towards RH reported in the main text.

Figure S4 reports the results obtained while applying Test2 on the MLG-based chemi-resistor (see main text).

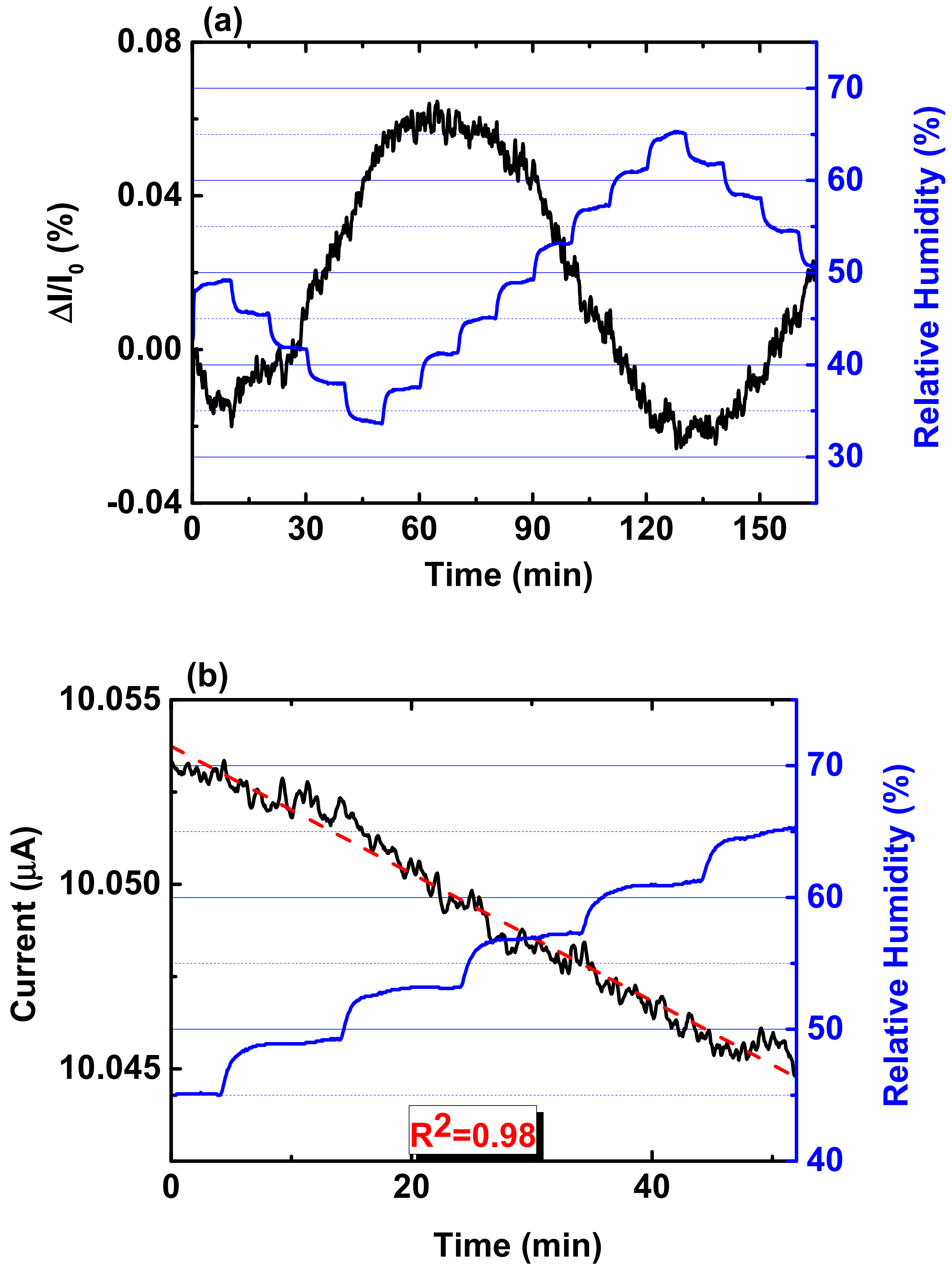
(a)
0.08
0.04
0.00
-0.04
ΔI/I0 (%)
70
60
50
40
30
Relative Humidity (%)
0
30
60
90
120
150
Time (min)
(b)
10.055
10.050
10.045
Current (μA)
70
60
50
40
Relative Humidity (%)
R2=0.98
0
20
40
Time (min)

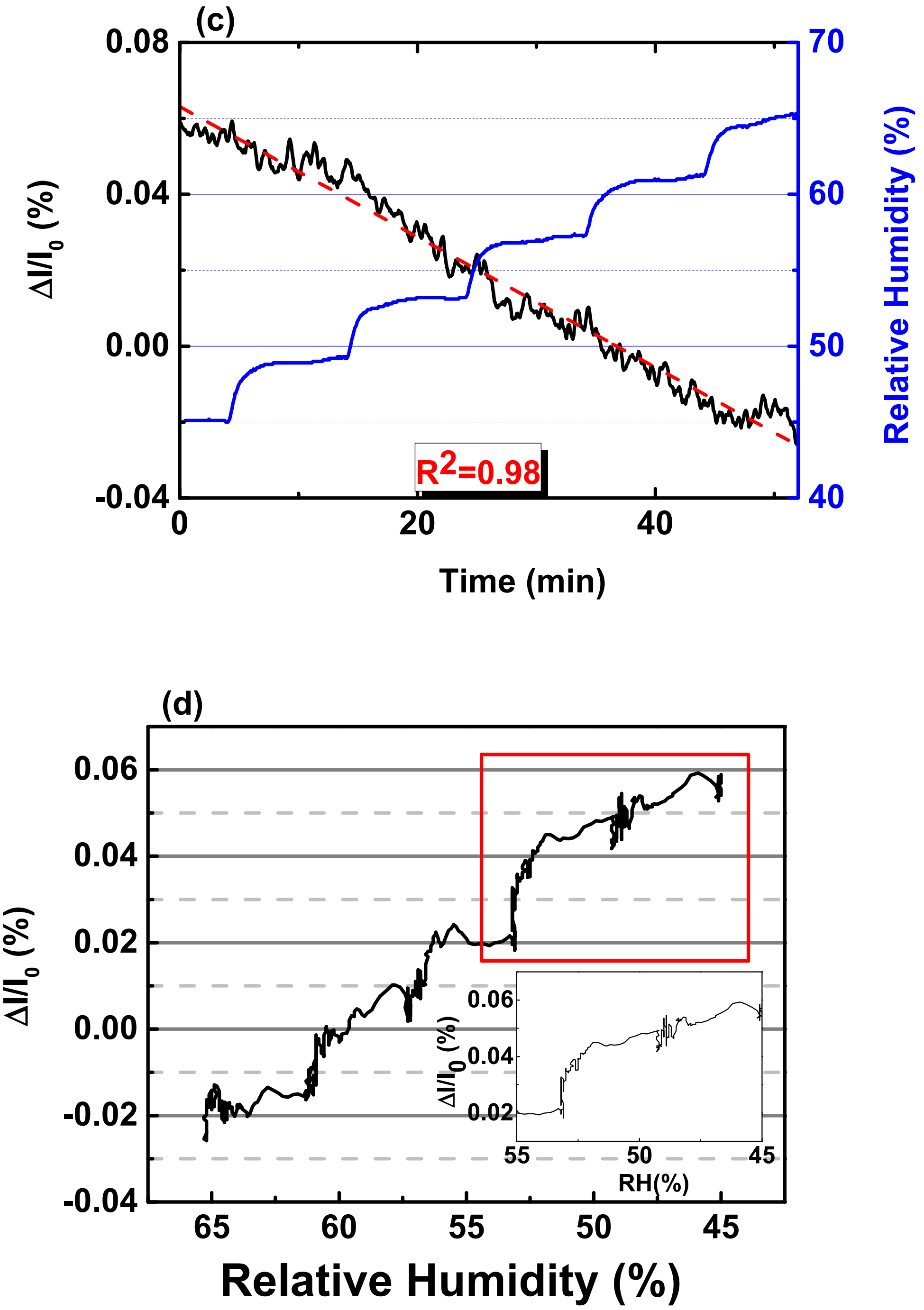

Figure 4. (**a**) Real-time behavior of the current variation (black line) upon Test2 (blue line). (**b**)–(**c**) Close-up of the current extracted from panel (**a**) before and after the normalization the normalization to I_0, respectively. The dashed red lines represent the fit of the current (black line). (**d**) $\Delta I/I_0$ plotted as a function of decreasing relative humidity (RH) level instead of as a function of time like in panel b. The x-axis is reversed to make easier the comparison with the panel (**c**). Inset. Zoom on the section of the plot enclosed in the red-box in panel (**d**).

Analogously to *Test1* reported in the main text, under the successive H_2O exposure steps (Figure S4a, blue line), $\Delta I/I_0$ plotted as a function of time (black line) never reaches a plateau along the exposure time of 10 min. On the contrary, $\Delta I/I_0$ shows a robust linear dependency from the humidity, as proven by the value of R^2 equal to 0.98 in the range 65%–45% of RH (Figure S4b,c). Considering the current recorded at the two extreme RH levels, we can estimate the sensitivity (S) as low as 0.4 nA/% (0.004%/%RH), that is consistent with the result obtained through Test1 (0.5 nA/%

or 0.005%/%RH if expressed as a percentage). Such a value further confirms the scarce sensitivity of MLG towards water molecules. The scarce sensitivity upon water molecules is further strengthened by the analysis of the output in the other two zones of the plot (Figure S4a). Decreasing the RH level from 50% down to 35% or from 65% down to 50% determines variations of about 0.02% or 0.04%.

By plotting the transient reported in Figure S4c as a function of RH level (Figure S4d), it can be seen that the maximum step-like variation of $\Delta I/I_0$ has height of about 0.04%/10%RH, as highlighted in the red box and enlarged in the Inset.

The findings hereby reported strongly support the achievements in the main text, regardless the adopted protocol.

Figure S5 reports the close up of the linear zones, obtained while applying Test3 on the MLG-based chemi-resistor (see main text).

From panel (a)–(b), we estimated the sensitivity to be roughly equal to 0.7 and 0.4 nA/% during the ramp down and ramp up of the protocol, respectively. From panel (c)–(d) we determined that the percentage variation of the current corresponding to the previous two panels is 0.01% and 0.008%/%RH.

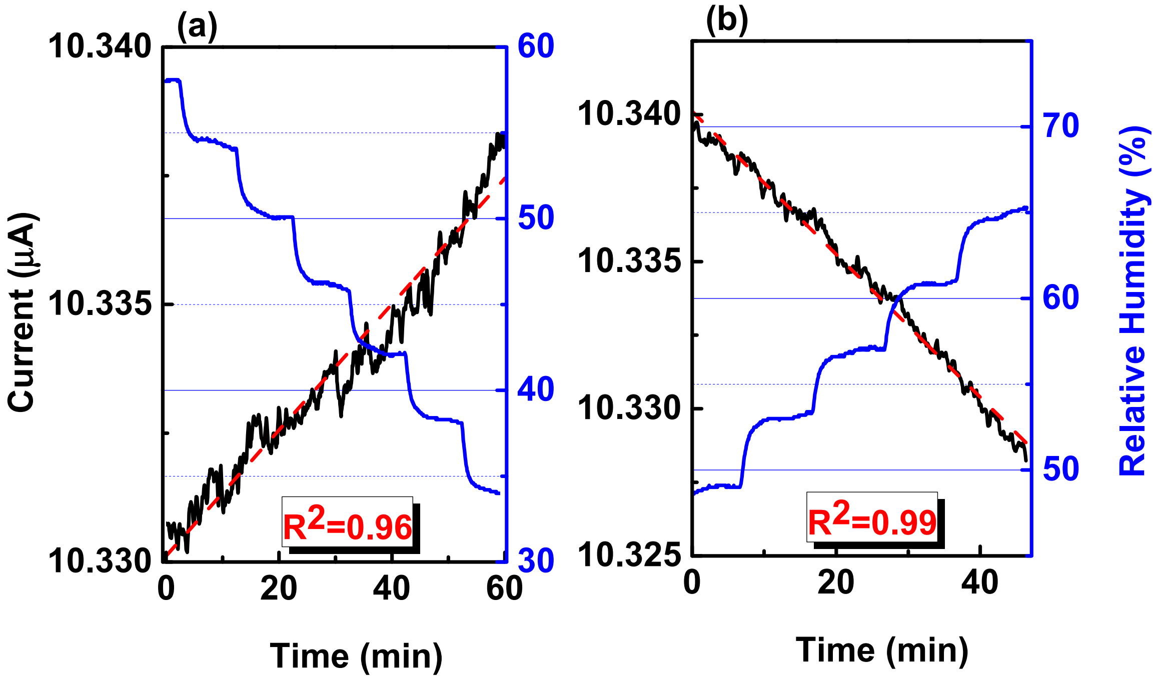

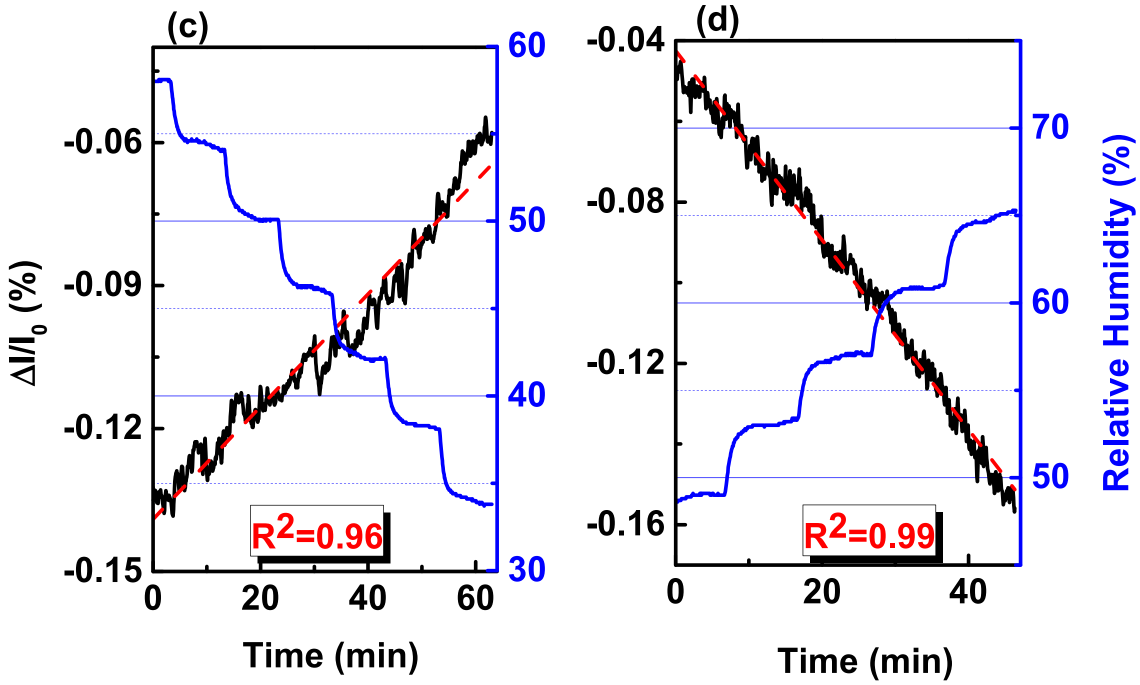

Figure 5. Close up of real-time behavior of (**a**)–(**b**) current and (**b**)–(**c**) current variation (black line) related to Figure 5a (main text). The values of the current are extracted from Figure 5a before the normalization to I_0. Left or right panels show the output while decreasing or increasing the RH level (blue line), respectively. The dashed red lines represent the fit of the outputs (black line).

The transient behavior of $(I-I_0)/I_0$ is reported in Figure S6a, where I_0 and I are the values of the current recorded at the inlet and along the exposure at the water vapors, respectively. Figure S5b shows the linear region of $\Delta I/I_0$ extracted from panel (a).

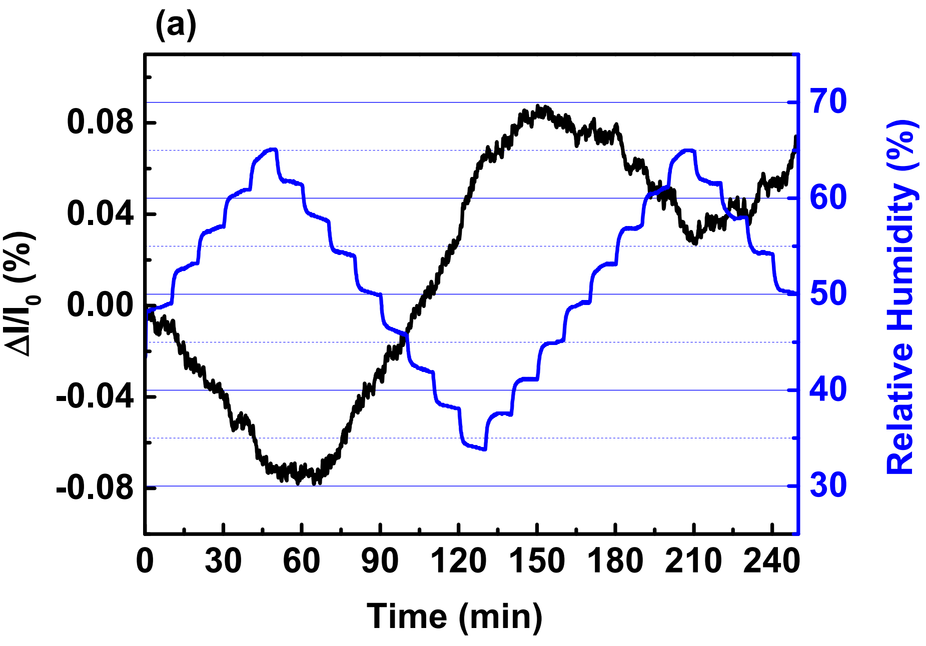

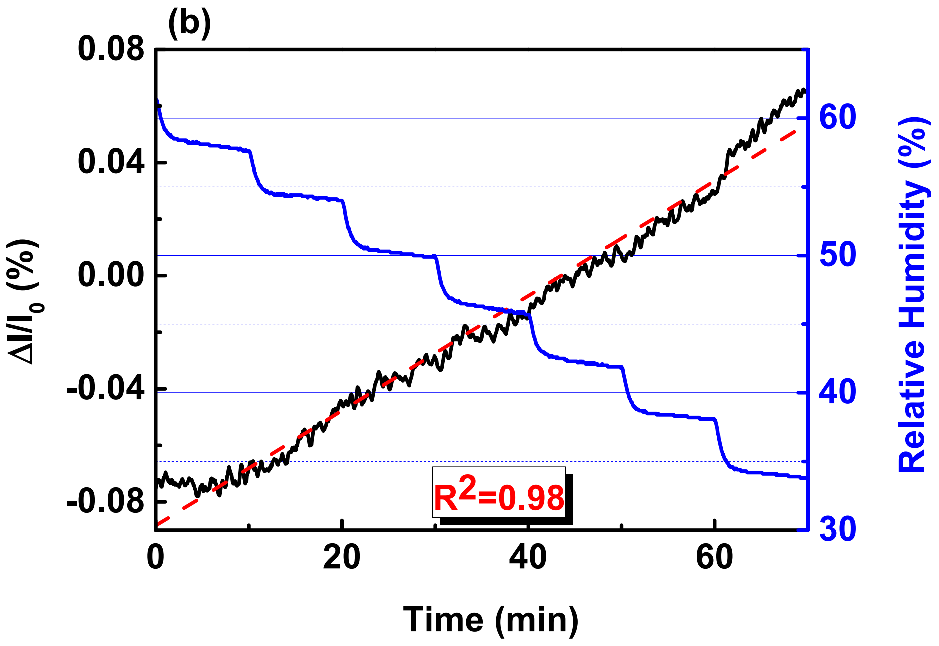

Figure 6. (**a**) Real-time behavior of the current variation (black line) upon Test3 (blue line). (**b**) Close-up of the output extracted from panel (**a**) while decreasing RH level (blue line). The dashed red line represents the fit of the current (black line).

The value of R^2 equal to 0.98 (Figure S6b) definitively confirms that the variation of current varies linearly with RH level in the range 30%–60%. The corresponding calculated sensitivity equals 0.005%/%RH, which value is well consistent with the findings determined from the first device and reported in the main text.

Finally, due to the achievements reported here and in the main text, where the highest variation of the signal was 0.01%/%RH, we can speculate that beyond the investigated RH range the sensitivity cannot overtake 0.02%/%RH.

© 2020 by the authors. Licensee MDPI, Basel, Switzerland. This article is an open access article distributed under the terms and conditions of the Creative Commons Attribution (CC BY) license (http://creativecommons.org/licenses/by/4.0/).
